# Genetic Alterations in Childhood Acute Lymphoblastic Leukemia: Interactions with Clinical Features and Treatment Response

**DOI:** 10.3390/cancers13164068

**Published:** 2021-08-12

**Authors:** Shawn H. R. Lee, Zhenhua Li, Si Ting Tai, Bernice L. Z. Oh, Allen E. J. Yeoh

**Affiliations:** 1 VIVA-University Children’s Cancer Centre, Khoo-Teck Puat-National University Children’s Medical Institute, National University Hospital, Singapore 119074, Singapore; paelhrs@nus.edu.sg (S.H.R.L.); paeolzb@nus.edu.sg (B.L.Z.O.); 2 Department of Pediatrics, Yong Loo Lin School of Medicine, National University of Singapore, Singapore 119074, Singapore; paeliz@nus.edu.sg (Z.L.); t.siting@nus.edu.sg (S.T.T.)

**Keywords:** childhood acute lymphoblastic leukemia, genetic subtypes, RNA-Seq, NCI criteria, MRD, *IKZF1^del^*

## Abstract

**Simple Summary:**

The latest molecular taxonomy of acute lymphoblastic leukemia (ALL) comprises >20 distinct genetic subtypes, each with their own unique clinical and prognostic characteristics. In this review, we describe how these new genetic subtypes interact with clinical presenting features, *IKZF1^del^*, treatment response, and outcomes, which is helpful for clinical use.

**Abstract:**

Acute lymphoblastic leukemia (ALL) is the most common cancer among children. This aggressive cancer comprises multiple molecular subtypes, each harboring a distinct constellation of somatic, and to a lesser extent, inherited genetic alterations. With recent advances in genomic analyses such as next-generation sequencing techniques, we can now clearly identify >20 different genetic subtypes in ALL. Clinically, identifying these genetic subtypes will better refine risk stratification and determine the optimal intensity of therapy for each patient. Underpinning each genetic subtype are unique clinical and therapeutic characteristics, such as age and presenting white blood cell (WBC) count. More importantly, within each genetic subtype, there is much less variability in treatment response and survival outcomes compared with current risk factors such as National Cancer Institute (NCI) criteria. We review how this new taxonomy of genetic subtypes in childhood ALL interacts with clinical risk factors used widely, i.e., age, presenting WBC, *IKZF1^del^*, treatment response, and outcomes.

## 1. Introduction

The most common type of cancer in children is acute lymphoblastic leukemia (ALL). Despite being one of the most curable cancers, with 5-year overall survival (OS) exceeding 90% with contemporary protocols [1], ALL remains a leading cause of cancer-related death in children and young adults [2].

**Figure 1:**
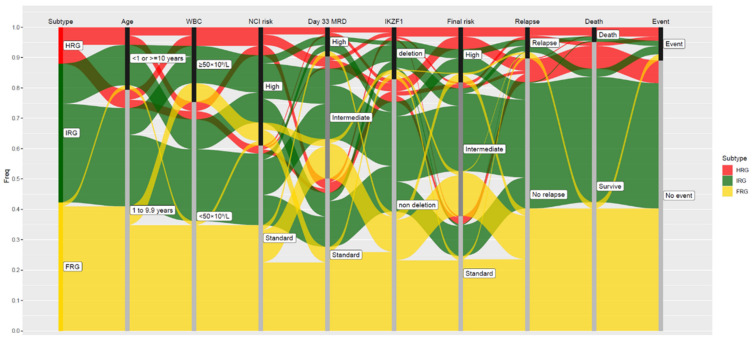
Interactions of genetic risk groups with various clinical prognostic risk factors and outcomes in childhood acute lymphoblastic leukemia (ALL). Each vertical stack in this Sankey diagram represents a prognostic factor or treatment outcome, with subcategories in different shades of black and gray. The relationships between various prognostic factors are demonstrated by flow lines, and the widths of the flow lines are proportional to the degree of interaction between genetics and clinical factors/outcomes. Favorable-risk subtypes are in shades of yellow, intermediate-risk subtypes are in shades of green, and high-risk subtypes are in shades of red. Day 33 MRD is categorized into three groups: standard, ≤0.01%; intermediate, 0.01% to 1%; high, ≥1%. Data are adapted from results of RNA-sequencing of children and adolescents with ALL in the Malaysia-Singapore cohort. Abbreviations: WBC, white blood cell; NCI: National Cancer Institute; MRD: minimal residual disease; HRG, high-risk genetics; IRG: intermediate-risk genetics; FRG: favorable-risk genetics.

Based on its lineage of origin, ALL can be broadly divided into B-lymphoblastic (B-ALL) and T-lymphoblastic leukemia (T-ALL), each harboring distinct constellations of somatic genetic alterations [3,4]. Advances in genomic analyses have enabled comprehensive interrogations of these genetic alterations, which in turn have improved the molecular taxonomy of ALL [5]. This deeper understanding of the molecular taxonomy of ALL has allowed us to further refine the current risk assignment beyond the conventional National Cancer Institute (NCI) criteria of age and white blood cell (WBC) count, and minimal residual disease (MRD). In this review, we focus on how NCI criteria, MRD, and *IKZF1^del^* interact with the new improved molecular taxonomy of ALL subtypes (Figure 1).

## 2. ALL Genetic Subtypes in 2020 and Beyond

Next-generation sequencing (NGS) technologies such as RNA-Seq, whole genome/whole exome sequencing (WGS/WES), and multiplex ligation probe-dependent amplification (MLPA) are increasingly used to define genetic subtypes. Using RNA-Seq, investigators from the Malaysia-Singapore (Ma-Spore) ALL study group could define >20 genetic subtypes of ALL, each with its own distinct genetic driver (Table 1).

Individually, these 20 genetic subtypes have their own distinct clinical characteristics, patterns of MRD response, and treatment outcomes. Based on their reported 5-year cumulative risk of relapse (CIR) and recommended treatment intensity, in this review we grouped them into three genetic risk groups: favorable- (FRG), intermediate- (IRG), and high-risk genetics (HRG) (Figure 1).

### 2.1. Favorable-Risk Genetics (FRG) Group

**Figure 2:**
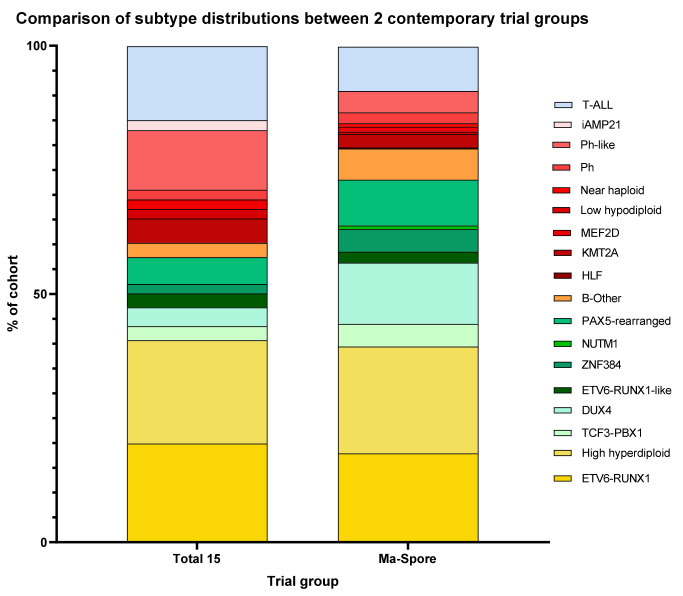
Spectrum of molecular subtypes of childhood ALL in the St Jude Total 15 cohort in the USA and the Malaysia-Singapore (Ma-Spore) cohort in Asia. The bar charts depict the estimated frequencies of each subtype of ALL among patients treated in these two frontline trials, updated with the current taxonomy of novel genetic abnormalities. The distribution profile differs slightly between these two cohorts, which is plausibly race-related, e.g., in proportions of *DUX4*, Ph-like, or T-ALL. Among the B-ALL subgroup, favorable-risk subtypes are in shades of yellow, intermediate-risk subtypes are in shades of green, and high-risk subtypes are in shades of red; B-Other is noted in orange; T-ALL is represented separately in blue. Data from Total 15 are reproduced with permission from Pui et al. Nat Rev Clin Oncol 2019. The prognosis of genetic subtypes possibly varies slightly between trial groups due to differences in risk stratification and treatment intensity, e.g., currently *DUX4* is now regarded by some trials (such as the recent St Jude Total 16 trial) as favorable-risk.

The favorable-risk genetics (FRG) group is defined as subtypes with <10% 5-year CIR (Table 1) and where de-intensification of therapy is safe and possible. This FRG group comprises two major classical subtypes: (1) *ETV6-RUNX1* and (2) Hyperdiploid >50 chromosomes. These two subtypes can usually be defined by conventional cytogenetics and RT-PCR. In addition, using the gene expression level of every gene on each chromosome, RNA-Seq can perform digital karyotyping by identifying the ploidy status of each chromosome. FRG accounted for ~40% of both the Ma-Spore and St Jude Total 15 cohorts (Figure 2).

For FRG in the Ma-Spore cohort, 60% of patients were NCI standard risk (SR). Of the patients in FRG, 45% achieved end-of-induction (EOI)-MRD negativity, while 37% were EOI-MRD IR (i.e., EOI MRD ≥0.01% and <1%) and 12% EOI-MRD HR (i.e., EOI MRD ≥ 1%). In FRG, only EOI-MRD HR and Ma-Spore HR criteria conferred a poorer event-free survival (EFS). Otherwise, all other criteria including NCI criteria, EOI-MRD SR and IR response, and treatment on Ma-Spore SR and IR arms did not significantly affect FRG survival. FRG had excellent 5-year CIR, EFS, and OS rates of 5%, 94%, and 100%, respectively. Furthermore, relapses in FRG are usually highly salvageable.

In the Children’s Oncology Group (COG), NCI SR with FRG are called SR-low risk. In this COG SR-low risk group, intensification through additional doses of PEG-asparaginase did not improve outcome [6]. In the Ma-Spore ALL 2010 (MS2010) trial, FRG who were also EOI-MRD-negative had exceedingly good outcomes using an anthracycline-free chemotherapy protocol. Similar to Ma-Spore, the UKALL2003 reported that only FRG with high EOI MRD ≥0.1% had poorer 5-year CIR >10% [7]. Therefore, in the ongoing prospective MS2020 study, FRG will receive de-intensified therapy even with a low positive EOI MRD (<0.1%). FRG will receive a more intensified therapy only if EOI MRD (≥0.1%), or if subsequent MRD timepoints are positive.

Although *TCF3-PBX1* and *DUX4* have low 5-year CIR (<10%), most trial groups (including us in the Ma-Spore trial group) kept them stratified as intermediate-risk genetics (IRG). Traditionally, *TCF3-PBX1* had poor treatment outcomes with less intensive therapy (see below). Because of these previously poor results, de-intensification of therapy for *TCF3-PBX1* carries significant risk. As such, despite a low CIR of <10%, we continue to regard *TCF3-PBX1* as IRG. *DUX4* patients also continue to be regarded as IRG, because they tended to have high PCR-based EOI MRD. For this reason, the majority of DUX4 patients were treated in IR and HR groups in the MS2003 [8] or MS2010 [9] studies, and even had hematopoietic stem cell transplant (HSCT) in first complete remission (CR) (see below).

### 2.2. Intermediate-Risk Genetics (IRG) Group

IRG, which accounts for ~45% of B-ALL, is the most genetically diverse group, comprising >10 different genetic subtypes (Table 1). Most IRG subtypes have an intermediate 5-year CIR of 10–20%. There are three exceptions with good outcomes: (1) *TCF3-PBX1*, (2) *DUX4,* and (3) *ZNF384*-rearranged (*ZNF384-r*). The remaining IRG are separately grouped based on similarity in gene expression profiles (GEP) or other rare genomic alterations. Despite their similar GEP, members of each group can be molecularly diverse and can have various distinct fusion partners. Individual fusion partners are rare by themselves, although most have been reported recurrently. Because of this rarity, we are unable to do further risk assignment based on the fusion partner. Within IRG, *DUX4* forms the largest genetic subgroup, with *PAX5*alt as the second largest group.

In Ma-Spore trials, almost 50% of IRG had an intermediate MRD response: 50% were MRD-positive at EOI but became MRD-negative by end-of-consolidation (EOC). The 5-year CIR ranged from 12.7% in *ETV6-RUNX1*-like to 20.7% in the B-others group (Table 1).

T-ALL lacks genetic prognostically distinct groups and has an intermediate 5-year CIR. The outcome of T-ALL has improved significantly with contemporary ALL therapy. In COG AALL0434 and MS2003, T-ALL outcomes have now approached that of B-ALL [10].

### 2.3. High-Risk Genetics (HRG) Group

The high-risk genetics (HRG) group is also genetically heterogeneous. This group accounts for around 15% of B-ALL, amongst which *KMT2A* rearrangements (*KMT2A*-r) and *BCR-ABL1* each account for ~3%. RNA-Seq has transformed the classification of the “B-others” category by conventional genetics. Specifically, using RNA-Seq, we can now define 13 distinct genetic subtypes in this conventional B-others group (Table 1). Of these 13 RNA-Seq genetic subtypes, five are especially high-risk: (1) *BCR-ABL1*, (2) *KMT2A*-*r*, (3) *MEF2D*, (4) *HLF-r*, and (5) *CRLF2*/*BCR-ABL1*-like ALL. These HRG are more prevalent in the USA, accounting for 12% of cases in the Total 15 cohort. In contrast, they occur in only 4% of the Ma-Spore patients (Figure 2). In MS2003/2010, patients in the HRG group have a high (20–55%) 5-year CIR.

## 3. Association of Subtypes with Race and Ethnicity

There are clear racial differences in the frequency of genetic subtypes of ALL. We compared the distribution of genetic subtypes between St Jude Total 15 and MS2003/2010 (Figure 2). The Total 15 cohort has predominantly white, black, and Hispanic races/ethnicities. In contrast, the Ma-Spore cohort is predominantly Chinese, Indian, and Malay. Subtle but important differences exist in the profile of genetic subtypes between these two cohorts, for example in the proportions of *DUX4* (4% in St Jude vs. 12% in Ma-Spore), *BCR-ABL*-like (12% in St Jude vs. 2% in Ma-Spore), *PAX5alt* (4% in St Jude vs. 9% in Ma-Spore), and T-ALL (15% in St Jude vs. 9% in Ma-Spore). It is plausible that the differences shown are closely related to the differences in races/ethnicities represented in these two cohorts. Even within the Ma-Spore cohort, notable differences exist amongst the major races. *CRLF2*, *ETV6-RUNX1*, and T-ALL appear more frequently in Indians, whilst *BCR-ABL1* is associated with the Malay race, and *ZNF384* and *TCF3-PBX1* are more common in the Chinese [11].

A meta-analysis of several studies identified that the frequency of *ETV6-RUNX1* fusion in childhood ALL in the Far East (Japan, Korea, Hong Kong, Singaporean Chinese, and Taiwan) was significantly lower than in the West (USA, Germany, Italy, France, and Chile) (15% vs. 22%). Similarly, the frequency of hyperdiploidy in East Asian children was also lower than Western children (15% vs. 32%) [12]. *TCF3-PBX1* and T-ALL are more frequently identified among African American children compared with other races [13,14,15,16]. Children of Hispanic/Latino ethnicity have a higher prevalence of Ph-like ALL, due to a higher prevalence of *CRLF2* rearrangements [17]. In fact, it is shown that multiple germline single nucleotide polymorphisms predisposing to the development of specific ALL subtypes differ by genetic ancestry, which may account for these subtypes’ differing prevalence by race/ethnicity, e.g., *GATA3* for Ph-like ALL, *BCL11A* for *TCF3-PBX1*, etc. [18,19,20]. Overall, these racial disparities are usually inferred by comparison across different study groups, and race is usually self-reported. These ethnic differences in biology plausibly contribute to the gap in survival outcomes between racial groups [14,15,16], and therefore should be investigated comprehensively in a large cohort using a more objective racial delineation.

## 4. Association of Genetic Subtypes with NCI Criteria of Age and WBC Count

The simplest and most widely used risk assignment criteria in childhood ALL are the NCI criteria, which consist of age and WBC count at presentation [21]. Because the NCI criteria can be consistently reported by any group, they are very useful as a standard comparative tool to compare the treatment outcomes between hospitals and cooperative trials. Infant or adolescent age (<1 or ≥10 years), or high WBC count at diagnosis (≥50 × 10^9^/L) is deemed NCI HR, which carries a worse prognosis than NCI SR patients [5,21]. NCI HR patients comprise up to a third of the Ma-Spore cohorts, where despite using MRD for risk assignment, NCI criteria remain highly prognostic [8].

**Figure 3:**
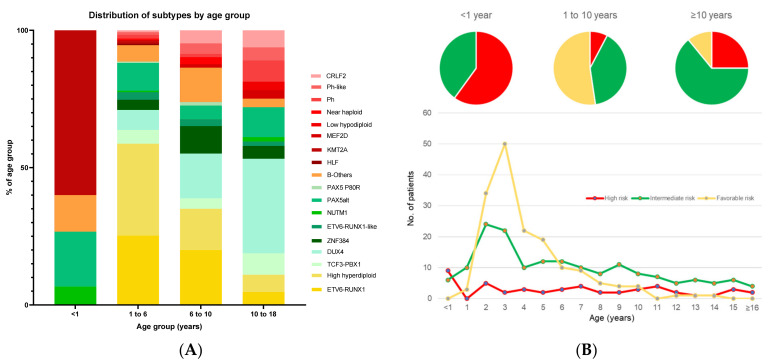
Association of B-cell acute lymphoblastic leukemia (B-ALL) subtypes with age. (**A**) Distribution of genetic subtypes by age group, and (**B**) distribution of genetic risk groups across ages in years. In (**A**), the proportions of individual subtypes within each age group are plotted, with subtypes summing to 100% in each category. In (**B**), the numbers of patients in each genetic risk group are plotted by age. Favorable-risk subtypes are in shades of yellow, intermediate-risk subtypes are in shades of green, and high-risk subtypes are in shades of red. B-others is depicted in orange. Here, the infant group overwhelmingly had *KMT2A* rearrangements with no favorable-risk genetics. This predominance of the high-risk genetics group in infants accounts for its poor outcome. After infancy, in ages 1–6 years, there is a striking peak of favorable-risk subtypes, such as *ETV6-RUNX1* and high hyperdiploidy. Subsequently, the proportion of high-risk subtypes increases with age, and the converse occurs with favorable-risk subtypes. Favorable-risk genetics is rare in adolescents, where there is a predominance of intermediate- and high-risk genetics. Data are adapted from results of RNA-sequencing of children and adolescents with B-ALL in the Malaysia-Singapore cohort.

**Figure 4:**
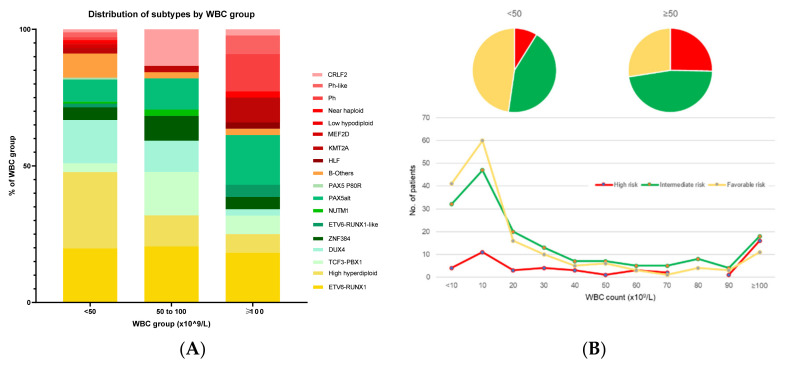
Association of B-cell acute lymphoblastic leukemia (B-ALL) subtypes with presenting WBC count. (**A**) Distribution of subtypes by WBC group, and (**B**) distribution of risk groups across WBC counts. In (**A**), the proportions of individual subtypes within each WBC group are plotted, with subtypes summing to 100% in each category. In (**B**), the numbers of patients in each risk group are plotted by WBC count and the distribution is as shown. Favorable-risk subtypes are in shades of yellow, intermediate-risk subtypes are in shades of green, and high-risk subtypes are in shades of red. B-others is depicted in orange. In general, favorable-risk or intermediate-risk subtypes are associated with low presenting WBC count (<20 × 10^9^/L), whilst higher-risk subtypes tend to present with a higher WBC, particularly Ph-like ALL, which tends to present with WBC >100 k. For the WBC < 50 k group, favorable- and intermediate-risk genetics predominate, accounting for their favorable outcomes. Data are adapted from results of RNA-sequencing of children and adolescents with B-ALL in the Malaysia-Singapore cohort. Abbreviations: WBC, white blood cell count.

The reason for these seemingly simple NCI criteria remaining as a strong prognostic factor is because ALL molecular subtypes are tightly associated with age and WBC (Figure 3 and Figure 4) [3,4]. Specifically, for children ≤1 year old and ≥10 years old, IRG and HRG are predominant (Figure 3). Similarly, in the group with presenting WBC ≥ 50 × 10^9^/L, IRG and HRG are also predominant (Figure 4). Taken together, NCI SR patients account for 60% of FRG, 52% of IRG, but only 22% of the HRG group, respectively. Conversely, NCI HR patients account for 40% of FRG, 48% of IRG, and 78% of the HRG group, respectively.

### 4.1. Infant ALL

Infants (<1 year of age) with ALL have the poorest overall treatment outcomes, which have not improved significantly in recent decades [22,23]. They are uniformly NCI HR because of their age of presentation, and they usually also have high WBC at presentation. *KMT2A* (*MLL*) rearrangements, in particular the t(4;11)(q21;q23) translocation, are most frequent in infants [24]. *KMT2A-r* ALL has been found to be over-represented in infants (80% of infants) compared with only 3–5% in older children [25]. Patients with t(4;11) generally present with hyperleukocytosis, with a high median WBC count >100,000/µL [22,25,26]. WBC count >300,000/µL confers particularly dismal prognosis [22,27]. Diagnosis at younger than 6 months was associated with poorer outcome in multivariable analyses in consecutive Interfant trials [22,23]; congenital ALL (diagnosis within first month of birth) was particularly dismal [28].

### 4.2. Adolescent and Young Adult ALL (AYA > 10 Years Old)

In general, fewer adolescents and young adults (AYA) develop ALL; however, AYA tend to have poorer outcomes. AYA patients comprised ~20% of the Ma-Spore cohort, amongst which only 11% were FRG. The tight inverse correlation between AYA and FRG explains why AYA have poorer outcomes (Figure 3). In MS2003/2010, AYA with FRG had similar 5-year EFS and OS to those in the age group of 1–10 years, suggesting that it is not the age that matters but the genetic subtypes represented in that age group.

## 5. Association of Genetic Subtypes with MRD and Outcomes

The quantitation of submicroscopic levels of disease in post-treatment bone marrow samples that are not visible by light microscopy is known as minimal residual disease (MRD). MRD is probably the strongest prognostic factor in ALL [29]. The two reasons why MRD is highly prognostic is because it is (1) very accurate in quantifying the risk of relapse and (2) highly informative (>90% patients have at least one marker). For example, PCR-based MRD used in Ma-Spore and Europe is informative in 90% of patients while flow-based MRD used in the USA is informative in 95% of patients. Currently, the level of MRD negativity is defined as 0.01% (or 1 in 10,000 cells). With the advent of NGS, sensitivity reaches 0.001% (1 in 100,000 cells) or better, and also allows for monitoring of all tumor-related sequences simultaneously. This extreme sensitivity allows for an even more refined risk stratification, by being able to identify a truly favorable group (i.e., those with truly extremely low or no disease), as well as being able to pick up early clonal evolution (new and low levels of disease) and possible eventual relapse [30,31,32].

Post-treatment MRD sums the combined effects of three critical aspects of ALL treatment in determining outcome: (1) genetic subtype, (2) effectiveness of chemotherapy given, and (3) host genetics affecting drug metabolism. For this reason, MRD is the strongest prognostic factor in ALL. However, because MRD is the combined effect of three factors, it is still dependent on genetic subtypes in predicting relapse.

Previously, cytogenetics and oncogene fusion screening (mainly *ETV6-RUNX1*, *TCF3-PBX1*, *BCR-ABL1,* and *KMT2A-AF4*) were informative in characterizing genetic subtype in only 50% of patients. Yet despite being informative in only half of patients, conventional genetic subtypes are highly prognostic, even in the era of MRD. Using RNA-Seq, we can now assign a specific genetic group for up to 93% of patients. With information on risk of relapse in 93% of patients, RNA-Seq genetic subtype may probably be more informative than MRD. Because RNA-Seq subtype is available early (usually before EOI), necessary alternative intervention (e.g., intensification, immunotherapy, etc.) can be brought forward. In addition, for the *ABL*-class fusion group, early use of a tyrosine kinase inhibitor, such as imatinib, dasatinib, or even ponatinib, can improve complete remission rates and outcomes. 

**Figure 5:**
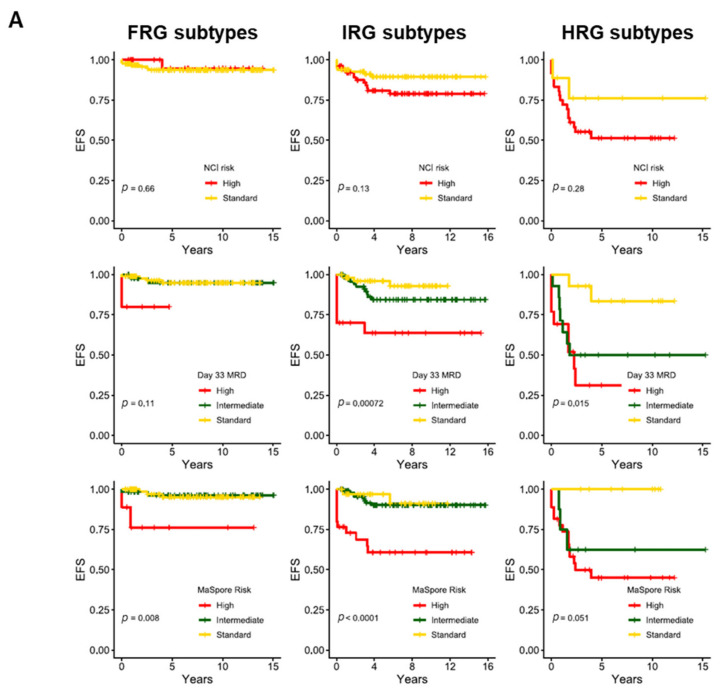

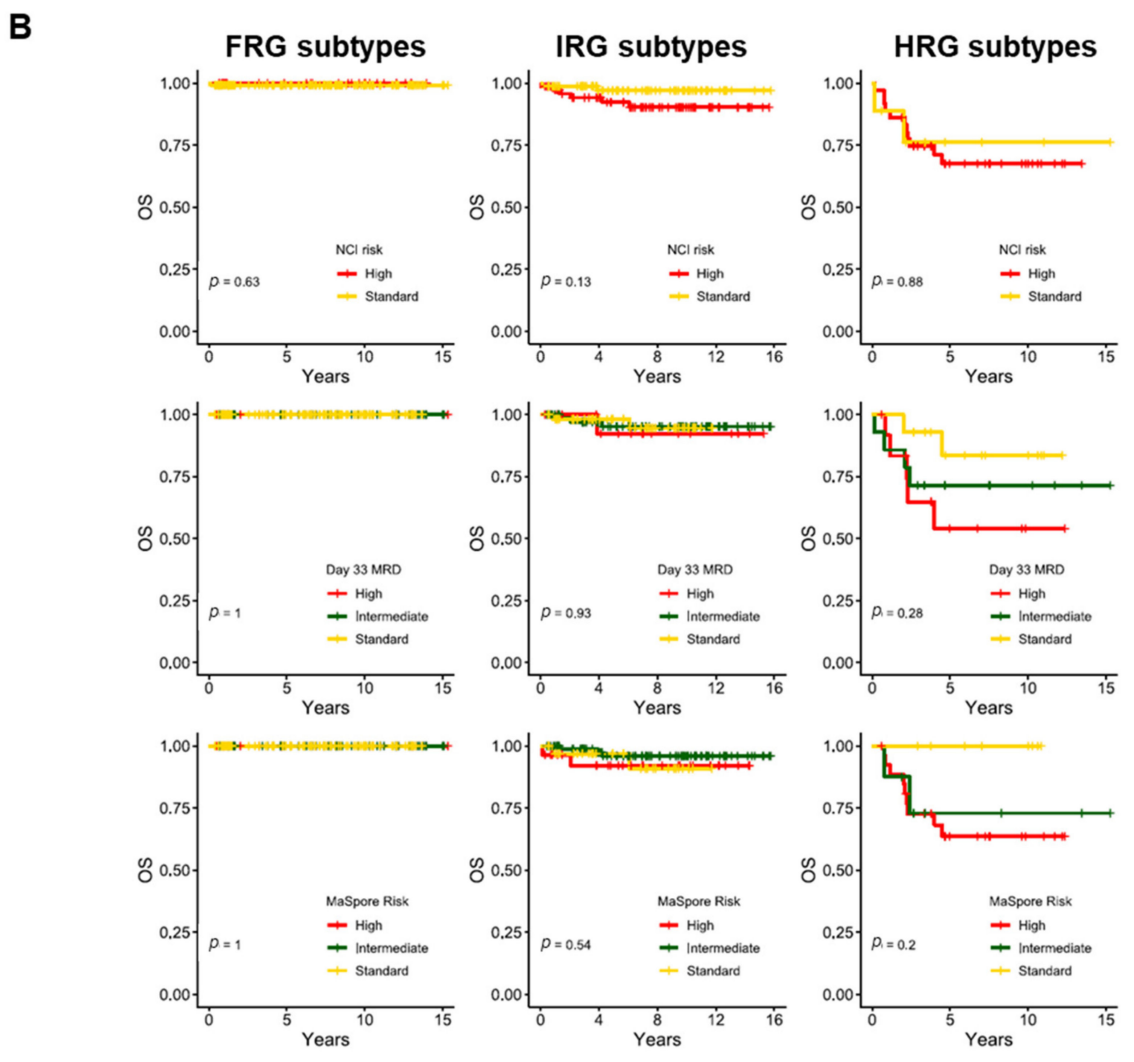
Treatment outcomes for genetic subtype risk groups with additional stratification by various prognostic markers in the Malaysia-Singapore cohort. (**A**) Event-free survival, and (**B**) overall survival. Genetic subtypes are defined by RNA-sequencing, and risk stratification is based on results from Malaysia-Singapore ALL trials. Favorable-risk (FRG) subtypes include hyperdiploidy and ETV6-RUNX1. Intermediate-risk (IRG) subtypes include DUX4, TCF3-PBX1, ETV6-RUNX1-like, ZNF384, ZNF384-like, PAX5alt, PAX5 P80R, IGH-CEBPE, NUTM1, and B-Others. High-risk subtypes (IRG) include TCF3-HLF, BCR-ABL, BCR-ABL-like, hypodiploidy, near-haploid, MEF2D, CRLF2, and KMT2A rearrangements. Ma-Spore risk classification integrates NCI risk group and Day 33 MRD. In the low-risk subtypes, outcomes are exceedingly favorable throughout, regardless of other prognostic factors. Conversely, these other factors further delineate the prognosis of higher-risk subtypes, especially with regards to EFS, highlighting the interplay between subtypes and these factors, and also underscoring the importance of integrating all these factors into risk stratification.

In the HRG group of the Ma-Spore cohorts, MRD remained prognostically important (*p* = 0.015 for EFS, *p* = 0.28 for OS, Figure 5). HRG patients who are EOI MRD-negative, when treated on the Ma-Spore HR chemotherapy arm, do well without any need for HSCT. However, HRG patients who are EOI MRD-positive (MRD-IR or MRD-HR) fare poorly: their 5-year EFS is <50%. In MS2020, HRG patients who are both EOI and middle-of-consolidation (MOC, week 8) MRD-positive qualify for chimeric antigen receptor (CAR)-T cell therapy or HSCT in first CR.

For IRG, EOI MRD remained the most significant prognostic factor for EFS (*p* = 7.2 × 10^−4^). In particular, IRG patients who are EOI MRD HR (>1%) have dismal outcomes (5-year EFS 40%). In MS2020, IRG with both high EOI (MRD≥1%) and high MOC MRD (week 8 ≥0.1%) also qualify for CAR-T cell therapy or HSCT in first CR. IRG patients who are EOI MRD SR have good 5-year EFS of 90–95%, which is equivalent to FRG patients. Therefore, these children can probably be treated with de-intensified chemotherapy.

For the FRG group, even if patients had low EOI MRD positivity (<0.1%), their 5-year EFS remained excellent. For this reason, they also qualify for treatment de-intensification. Only in high EOI MRD (≥1%) is treatment intensification truly indicated. For the Ma-Spore trials, in the rare situation where EOC MRD remains high (≥0.1%), FRG patients qualify for CAR-T cell therapy or HSCT in first CR because these patients usually have a poorer 5-year EFS of ~75%.

Below, we summarize the specific clinical features associated with each subtype.

### 5.1. ETV6-RUNX1 and Hyperdiploidy

*ETV6-RUNX1* and hyperdiploidy both have excellent outcomes (Table 2). However, these subtypes do differ in their MRD response: *ETV6-RUNX1* has more rapid MRD clearance [33]. In the Total 15 study, 58% of the patients with *ETV6-RUNX1* fusion had MRD < 0.01% at Day 19, compared with 44% of hyperdiploid diseases [34]. Similarly, in Total 16, 54% of *ETV6-RUNX1* had MRD < 0.01% at Day 15 compared with 31% of hyperdiploid cases. The same trend is seen for COG [35,36], UKALL2003 [7], and MS2003/2010 [8,9]. Overall, both *ETV6-RUNX1* and hyperdiploid ALL have excellent outcomes with contemporary 5-year EFS and OS exceeding 90% [34,37,38]. In MS2003/2010, all *ETV6-RUNX1* and hyperdiploid cases received a de-intensified, three-drug, dexamethasone-based induction without anthracyclines, with excellent results.

### 5.2. PBX1 Fusions Including TCF3-PBX1

*TCF3-PBX1*, with t(1;19)(q23;p13) translocation, is more commonly found in children. *TCF3-PBX1* accounts for ~5% of childhood ALL, but only 1% in adults [3,39]. *TCF3-PBX1* generally presents with higher WBC (median 56,000/µL) [40,41]. *TCF3-PBX1* is also more common among African Americans. Rarely, other than *TCF3*, *PBX1* may fuse with another partner. These rare fusions involving *PBX1* and other partners have similar GEP as *TCF3-PBX1* and are classified together under the PBX1 fusion group.

Historically, in the era of lower-intensity therapy, *TCF3-PBX1* ALL had poorer outcomes [42]. With more intensive chemotherapy, the outcomes for *TCF3-PBX1* have improved considerably. In MS2003/2010, *PBX1* patients have a low 5-year CIR of 5.6%. Researchers in Hong Kong reported no relapse and 100% 5-year survival in 30 *TCF3-PBX1* patients treated from 1997 to 2016 [43].

The UKALL group reported that *TCF3-PBX1* has the most rapid clearance of MRD [33], with >85% of patients having an EOI MRD level ≤0.01% [44,45]. In MS2003/2010, where most patients received three-drug induction, *TCF3-PBX1* patients achieved MRD negativity in 53% and 94% of cases by EOI and EOC, respectively. However, in *TCF3-PBX1*, failure to achieve MRD negativity by EOC predicts a poorer outcome.

In Total 15, where cranial irradiation is omitted, the St Jude investigators reported an increased risk of central nervous system (CNS) relapse (9.0 ± 5.1%) [46]. With two additional intensified intrathecal therapies during induction and PEG L-asparaginase intensification in Total 16, no *TCF3-PBX1* patients developed CNS relapse. The 5-year CIR of *TCF3-PBX1* in Total 16 was 6%, with an intermediate 5-year EFS and OS of 88% because of transplant-related mortality [47].

Overall, with sufficiently intensive therapy such as the medium-risk arm of ALL-BFM, *TCF3-PBX1* patients have excellent outcomes. In general, many groups such as Ma-Spore are de-intensifying therapy for EOI MRD-negative patients. Therefore, for *TCF3-PBX1* patients who are EOI MRD-negative, de-intensification of therapy can be attempted. However, despite its overall favorable outcome as a genetic subtype, de-intensifying therapy for all *TCF3-PBX1* patients may be risky. This is because of the historically poorer outcomes of *TCF3-PBX1* with less intensive regimens, and also because relapses of *TCF3-PBX1* are difficult to salvage even with HSCT. It is not clear yet whether CAR-T cell therapy will be able to effectively salvage *TCF3-PBX1* relapses. Although they have a low 5-year CIR of only 5.6% in MS2003/2010, we still recommend that *TCF3-PBX1* be classified as IRG. MS2020 will only de-intensify therapy for *TCF3-PBX1* patients who are EOI MRD-negative, which accounts for only 53% of the *TCF3-PBX1* cohort.

### 5.3. ZNF384-Rearranged (ZNF384-r)

*ZNF384-r* is found in 1–6% of childhood B-ALL and 5–15% of adult B-ALL cases [5,48]. It is found more frequently among older children [48,49], with a median age of 6.8 years in MS2003/2010. The median WBC of patients at presentation is slightly higher at 37,000/µL in MS2003/2010, similar to another report. This subtype also appears to be more common in Asians [50].

For *ZNF384-r*, MS2003/2010 reported a 5-year CIR of 6.3%, 5-year EFS of 83%, and 5-year OS of 93%, which is similar to the Ponte Di Legno group [51] with 5-year EFS of 84% and OS of 91%. However, in MS2003/2010, the kinetics of MRD response for *ZNF384*r is slow. Only 18% of *ZNF384* cases were EOI MRD-negative. This slow response improved by EOC, with 77% negative in EOC MRD. Treatment response and outcomes varied with different rearrangement partners of *ZNF384*. Patients with EP300-*ZNF384* ALL had better prednisolone response [50] and EFS than other *ZNF384*-rearranged cases [48]. Of note, the relapse of patients with *TCF3*-*ZNF384* and TAF15-*ZNF384* rearrangements can occur late, several years after the completion of treatment [48,50,52].

### 5.4. PAX5

*PAX5*-rearranged ALL comprises two genetic subgroups: *PAX5*-P80R and, more commonly, *PAX5*alt [53]. These *PAX5*-rearranged cases are usually older. Gu et al. found that the median ages at diagnosis for *PAX5*-P80R and *PAX5*alt ALL subjects were 22.0 years and 15.4 years, respectively [3]. The proportion of *PAX5*-P80R cases increased with age even into adulthood, while *PAX5*-alt cases peaked at adolescence [3]. *PAX5*alt has higher presenting WBC (>50,000/µL). In fact, this effect seemed to be additive, where patients showing more than one *PAX5* aberration had an ever higher WBC count compared with patients with only one *PAX5* abnormality [53].

Gu et al. reported that ~70% *PAX5*alt patients achieved EOI MRD < 0.01%, indicating a relatively good response to treatment [3]. However, in children, the outcome in COG AALL0232 is only intermediate, with OS of ~75%. In adults, the outcome is poor, with OS of 42%. By comparison, the *PAX5* P80R subtype generally responds rapidly to therapy, with >90% of patients achieving MRD <0.01% at EOI [3], although outcomes vary in different studies. In a German cohort reported by Bastian et al. with both pediatric and adult patients, this subtype had an OS of 80% [54]. In adults, *PAX5* P80R ALL had a relatively favorable outcome compared with *PAX5*alt, with an OS of 62% [3].

In St Jude Total 16, *PAX5*alt had an intermediate outcome with a 5-year CIR, EFS, and OS of 17%, 83%, and 100%. In MS2003/2010, 39% of *PAX5*alt have negative EOI MRD. These *PAX5*alt patients who are EOI MRD-negative do well. However, we noticed a poorer outcome for *PAX5*alt who are *IKZF1^del^* (see Section 6 below).

### 5.5. ETV6-RUNX1-Like

More recently discovered is the *ETV6-RUNX1*-like subtype, accounting for ~3% of B-ALL. Despite a lack of *ETV6-RUNX1* fusion, such cases clustered with the *ETV6-RUNX1*-positive cases [55]. *ETV6-RUNX1*-like ALL seems to occur almost exclusively in children and adolescents, and presents at a similar median age to *ETV6-RUNX1* ALL at 3–5 years of age [3,55,56]. Similar to *ETV6-RUNX1*, *ETV6-RUNX1*-like ALL does not have elevated presenting WBC [38,56]. Surprisingly, although it has a similar GEP to *ETV6-RUNX1*, *ETV6-RUNX1*-like has poorer outcomes. In fact, in the recent Total 16 study, *ETV6-RUNX1*-like patients had amongst the highest relapse rates (5-year CIR 22%) [57], consistent with the poor outcomes from earlier reports [55].

*ETV6-RUNX1*-like patients commonly have *IKZF1^del^*. Because of the small numbers, it is unclear in MS2003/2010 whether *IKZF1^del^* conferred an adverse outcome for *ETV6-RUNX1*-like. In MS2003/2010, *ETV6-RUNX1*-like has 5-year CIR and OS of 13% and 89%, respectively. For this reason, *ETV6-RUNX1*-like may benefit from higher-intensity therapy.

### 5.6. DUX4

The *DUX4* subtype is characterized by the rearrangement of the *DUX4* gene to the IGH locus. This rearrangement brings *DUX4* close to the IGH enhancer Eμ, resulting in a distinctive GEP with exceedingly high expression of *DUX4*. It is also associated with transcriptional deregulation (usually deletion) of *ERG* and *IKZF1^del^* (63% and 28%, respectively) [58,59]. *DUX4* patients tend to be slightly older (median age 9.8 y in Ma-Spore) [60,61,62], with low white cell counts (median 10,000/µL) [60,63,64].

Of the IRG group, *DUX4* ALL has a very notably peculiar MRD response. In Total 16, where MRD was flow-based, all *DUX4* patients were MRD-positive at Day 15 of induction, with 50% having high MRD >1% [57]. However, by EOI (Day 42), 95% became MRD-negative. In Total 16, 40% of *DUX4* were treated on low-risk and 60% on standard-risk arms, and the outcomes for *DUX4* were excellent (5-year EFS 95% with no relapses). In contrast, in MS2003/20110 which used PCR MRD, 74% of *DUX4* were EOI (Day 33) MRD-positive, with 25% EOI MRD HR (>1%). In the AIEOP-BFM ALL 2009 study, around 90% of the *DUX4* patients had positive EOI or EOC MRD [65]. Despite this, *DUX4* in AIEOP-BFM 2009 also had very favorable outcomes. This favorable outcome is similar to MS2003/2010, where most patients were treated on the IR or HR arm, including HSCT in first CR. In MS2003/2010, the 5-year CIR, EFS, and OS were 9%, 91%, and 98%, respectively.

As the induction therapies for St Jude, AIEOP-BFM, and Ma-Spore are not very different, the discordance of EOI MRD is surprising and significant. We postulate that this difference in EOI MRD is because of differences in MRD detected by flow cytometry and PCR. Specifically, compared with PCR MRD, flow MRD is probably more adept at tracking the response of *DUX4* ALL. One plausible reason is the tendency for switching of *DUX4* leukemia clones to a monocytic lineage [66]. Flow MRD can detect and exclude these monocytic-switched cells. Since these monocytic-switched cells do not contribute to relapse, flow MRD is probably more accurate in quantifying the true leukemic MRD burden. On the other hand, PCR-based MRD cannot distinguish between these monocytic-switched cells and *DUX4* leukemia cells as they both carry the same clonal Ig/TCR marker. Taken together, we believe that PCR-based MRD may overestimate the potential of relapse of *DUX4* patients. In fact, for *DUX4*, we find that EOI MRD based on PCR Ig/TCR is not prognostic of outcome.

Interestingly, *ERG* deletion, which occurs almost exclusively in the *DUX4* subtype, is associated with better MRD response and outcome [67]. *IKZF1^del^* as no adverse effect on *DUX4*. Overall, most trial groups consistently report excellent results for *DUX4*, with EFS and OS usually exceeding 90% [3]. Although now considered by some trials to be favorable [57], due to seemingly poor MRD response, *DUX4* are often treated as high-risk [57,65]. Given their excellent outcomes, this raises the question as to whether these patients are actually over-treated. The possibility of de-intensifying therapy in this group needs to be examined carefully. To begin addressing this question prospectively, in MS2020, *DUX4* will be treated on the IR arm regardless of EOI MRD and will only undergo HSCT if MRD levels are rising despite chemotherapy.

### 5.7. Philadelphia (Ph, BCR-ABL1)-Positive

Philadelphia chromosome (Ph) ALL, with the *BCR-ABL1* fusion, is one of the quintessential high-risk ALL subtypes. It is defined by t(9;22). The incidence of Ph ALL increases with age >10 years; it accounts for 2% to 5% of childhood ALL but 25% of adult ALL. With standard 4-drug ALL induction therapy, there is an exceedingly high induction failure rate of 11% compared with the 2% to 3% seen among children with non-Ph ALL [68]. Historically, even with HSCT in first CR, EFS rates were dismal, ranging from 28% to 32% [69].

Tyrosine kinase inhibitors (TKIs) have dramatically changed the treatment landscape for Ph ALL, both in children and adults. The addition of imatinib to combination chemotherapy doubled EFS rates, compared with those who did not receive imatinib [70,71]. Recently, a large randomized trial in China comparing dasatinib and imatinib showed the superiority of dasatinib with 4-year EFS and OS rates of 71.0% and 88.4%, respectively, compared with 48.9% and 69.2%, respectively, for imatinib [72]. Notably, EOI MRD negativity of dasatinib and imatinib was similar. For Ma-Spore, the addition of imatinib to the high-risk chemotherapy backbone reduced the 5-year CIR of Ph ALL from 58% in MS2003 to 19% in MS2010. Although the toxicity of therapy also increased, the 5-year OS in MS2010 still improved significantly for Ph ALL.

In general, most trial groups regard Ph ALL as high-risk or very high-risk, and are treated on a Ph ALL-specific protocol. The European study groups such as UKALL [73,74], AEIOP-BFM [75], and DCOG [37] enrolled Ph ALL in separate protocol EsPhALL [70]. Similarly, the COG considered Ph ALL as “very high risk”, and enrolled these patients in the separate study AALL0031 [71]. Due to concerns about treatment-related mortality from intensive chemotherapy plus TKI, MS2020 will enroll Ph ALL on three-drug induction with a TKI (either dasatinib or imatinib). EOI MRD-negative Ph ALL patients will continue on TKI plus standard-risk, reduced intensity chemotherapy while EOI MRD-positive patients qualify for CAR-T or HSCT in first CR. MS2020 aims to use an intensive TKI on top of a less intensive chemotherapy backbone.

### 5.8. BCR-ABL-Like (Ph-like) with or without CRLF2 Rearrangements

*BCR-ABL*-like, or Ph-like, ALL is characterized by a spectrum of diverse genetic alterations and has a similar transcriptional profile to Ph-positive ALL but without the *BCR-ABL1* fusion [76]. The prevalence of Ph-like ALL increases significantly with age and NCI risk group, from 10% among SR children and 13% for HR children, to 21% among adolescents, and 27% among young adults [77]. While the prevalence of *BCR-ABL* ALL rises progressively with age, Ph-like ALL differs in that it peaks in young adulthood [5]. Both Ph and Ph-like ALL are usually associated with higher leukocyte counts at presentation [77,78,79].

Similar to Ph ALL, Ph-like ALL typically has high EOI and EOC MRD. Ph-like ALL also has higher rates of treatment failure compared with non-Ph-like ALL patients. For Ph-like ALL, the 5-year EFS and OS rates in children and AYA are 58% and 73% for children, and 41% and 66% for AYA, respectively [77,80,81]. Survival is particularly poor for Ph-like patients with elevated EOI MRD [80]. The higher prevalence of Ph-like ALL in AYA may partly explain the adverse outcomes in this age group.

Ph-like ALL is characterized by multiple genomic alterations, and the majority of alterations can be targeted effectively with ABL (e.g., dasatinib) or *JAK* inhibition (e.g., ruxolitinib). Currently, the inferior survival for Ph-like ALL appears to occur regardless of the underlying genomic alteration. A multiple combinatorial approach to chemotherapy with targeted therapies is currently being tested in frontline studies, giving further hope in the treatment of this high-risk subtype [82]. For MS2020, Ph-like ALL with ABL-class fusion will be treated with dasatinib or imatinib plus SR chemotherapy backbone. As RNA-Seq results may return only at the EOI, Ph-like ALL patients will qualify for HSCT or CAR-T therapy if MRD remains positive at week 8 (MOC).

Ph-like ALL is frequently associated with *CRLF2* rearrangements, and IGH-*CRLF2* rearrangement accounts for almost 50% of Ph-like ALL in AYA and adults [82]. Patients with *CRLF2* rearrangements had poorer treatment outcomes in general compared with those without. In particular, those with *CRLF2*-*PY2R8* rearrangements had the most inferior EFS (5-year EFS of 57% vs. 83% for other B-ALL) and significantly increased CIR (43% vs. 14% for other B-ALL) [17,83,84]. In the Ma-Spore studies, we chose to separate *BCR-ABL1*-like ALL into two distinct groups based on the presence of *CRLF2* expression, and on ABL-class fusion since it is targetable.

*CRLF2* rearrangements are most common in Ph-like and Down syndrome-associated ALL, but also occur without the transcriptional signature of Ph-like ALL [85]. *CRLF2* is overexpressed in approximately 15% of adult and high-risk pediatric B-ALL, and is associated with Hispanic ethnicity [85,86]. These rearrangements are age-dependent, with *P2RY8-CRLF2* associated with younger age (median 4 years) and *IGH-CRLF2* associated with older age (median 8 years) [87,88,89]. Although *CRLF2* rearrangements have not been found to be associated with WBC count at diagnosis in general [89], high-risk patients with *CRLF2* rearrangements had a higher median WBC than those without (92 × 10^9^/L vs. 60 × 10^9^/L) [17].

### 5.9. MEF2D

Myocyte enhancer factor 2D (*MEF2D*), another recently discovered subtype, is characterized by multiple fusion partners, the most common being *MEF2D*-*BCL*9. *MEF2D* rearrangement occurs in approximately 1–4% of B-ALL in children and 6–7% of adult ALL [1,3,90]. This subtype occurs more frequently in older children and adolescents (median of 9–14 years) [1,91,92]. These patients also usually have elevated WBC counts (median >20,000/mL) at presentation and, as a result, are mostly classified as NCI high risk [92].

Although *MEF2D*-rearranged ALL is uncommon (1% in Ma-Spore), they have an inferior outcome. An analysis of children enrolled on the AALL0232 study of high-risk pre-B ALL showed that the 5-year event-free survival (EFS) of *MEF2D*-rearranged ALL was 71.6%, compared with 87.3% for other pre-B ALL cases. However, in this same study, *MEF2D* rearrangements lost prognostic impact after correcting for age, sex, and WBC. In a smaller cohort of four patients with *MEF2D*-*BCL*9 rearrangement, all were noted to have chemotherapy resistance and very early relapse, with statistically significantly poorer EFS and OS rates for *MEF2D* patients [93]. Similarly, an analysis of a small cohort showed that although there was no poor steroid response associated with this subtype, there was a 53.3% relapse rate, all of whom died [92]. In Total 16, there were only three *MEF2D-r* ALL with 5-year CIR, EFS, and OS of 33%, 67%, and 67%, respectively. In MS2003/2020, all four *MEF2D-r* patients were alive and disease-free. Because it is uncommon, it is only retrospective pooling of a large number of *MEF2D*-rearranged cases by groups, such as the Ponte de Legno group, that allows us to accurately determine if they have poorer outcomes [91]. Increased expression of *MEF2D* is associated with activation of HDAC9 [90,91], which in turn may confer sensitivity to histone deacetylase inhibitor treatment [94], such as vorinostat, and proteasome inhibitor treatment, such as bortezomib [91].

### 5.10. KMT2A-Rearranged/MLL

*KMT2A*-rearranged ALL is a very high-risk disease with poor response to treatment. Disappointingly, there has not been much improvement in outcome for *KMT2A-r* ALL. The most recent studies have indicated only modest improvement (4-year EFS of 40–50% and OS of 50–55%), compared with 20–40% historically [22,27,95,96]. The recently published Interfant-06 study showed only ~20% and ~40% of patients achieved MRD negativity at EOI and EOC, respectively [95]. The poor outcomes are due to intrinsic resistance of *KMT2A-r* blasts, which often have (in vitro) resistance to important chemotherapeutic drugs such as prednisone and l-asparaginase, although they typically have acute sensitivity to cytarabine [97]. Although most patients (~80–90%) will go into remission initially, a high proportion (50–60%) of them will relapse, most commonly in the bone marrow [27].

In general, there is no significant association between relapse or survival in *KMT2A-r* ALL and any particular fusion partner. Therefore, most current clinical risk stratifications do not take the fusion partner into account [25,96,98]. Although HSCT plays a strong role in consolidation therapy for most high-risk leukemias and T-ALL, this is not the case for *KMT2A-r* ALL where HSCT has not yet been shown to be of benefit in general [99,100,101]. However, the Interfant-99 ALL trial identified a small subgroup of infants with additional poor prognostic factors where HSCT appeared to be valuable [22]. This subtype is universally stratified as high-risk to receive intensified therapy [35,36,37,73,74,75,102], or similar to Ph-ALL, where it is managed as separate protocols [95,103].

It is not clear whether CAR-T CD19 therapy will be a game changer for KMT2A-*r* ALL. This is because *KMT2A-r* ALL may relapse with monocytic switch and loss of CD19 expression.

### 5.11. Low-Hypodiploid and Near-Haploid

Near-haploid (24–30 chromosomes) and low-hypodiploid (31–39 chromosomes) are rare; they are each seen in ∼0.5% of childhood ALL [104,105,106,107]. An interesting frequent phenomenon in hypodiploid ALL is doubling of the chromosomal content, resulting in clones with 50 to 78 chromosomes, masquerading as masked hyperdiploidy [107]. Interestingly, near-haploidy has never been reported in adult ALL, whereas ~4% of cases harbor low hypodiploidy [105,107,108]. The age profiles of near-haploidy and low hypodiploidy differ, with the former being restricted solely to childhood/adolescence and the latter becoming more frequent with increasing age. All reported near-haploid ALL cases have been 1 to 19 years old at diagnosis, with a median age around 5 years. On the other hand, low hypodiploidy occurs at all ages and is characterized by an older pediatric age group with a median age of 13–15 years [108,109,110]. Both groups display relatively low white blood cell counts at diagnosis, with median blood counts usually <10 × 10^9^/L [105,106,109,110].

Similar to infant ALL, children with hypodiploid and near-haploid ALL have continued to fare poorly in recent decades. MRD response is generally unfavorable, with only 50% achieving EOI MRD <0.01% [109]. In terms of survival, the COG AALL0031 study demonstrated a 4-year OS rate of 54% for this group [103]. More recently, a multicenter retrospective study on 306 patients (representing 16 cooperative study groups) did not show much difference, with 5-year EFS of 55% and an OS rate of 61%, even with MRD-directed therapy [109]. Expectedly, a high proportion of patients had poor early response by morphologic examination and/or high MRD after induction therapy. Similar to infant ALL, transplantation did not improve outcome compared with chemotherapy alone, especially among the subgroup of patients who achieved a negative MRD status [104,111]. Near-haploid ALL is historically thought to have poorer outcomes than hypodiploid ALL, with EFS reported to be 20–40% [106,107], although evidence is conflicting with some trial groups reporting no difference between outcomes for hypodiploidy and near-haploidy [112]. The UKALL [73,74] and NOPHO [113] studies stratified this subtype as high-risk while COG included it as one of the very high-risk subtypes in the AALL0031 study [103].

In children, low-hypodiploid ALL is associated with TP53 germline mutation, which confers a poorer outcome regardless of genetic subtype [114].

### 5.12. HLF

This particular rare subtype portends an extremely poor prognosis; it is one of the subtypes that is regarded as almost incurable. This subtype has translocation t(17;19)(q22;p13), resulting in the fusion gene *TCF3-HLF*, which is typically associated with treatment failure, relapse, and death within two years from diagnosis [115,116,117]. Interestingly, in vitro studies show exquisite sensitivity of *TCF3-HLF* leukemic cells to the *BCL*2 inhibitor venetoclax (ABT-199), suggesting a new therapeutic option for this otherwise fatal subtype [117]. Additionally, a recent report has described the successful use of CD-19 directed immunotherapy with blinatumumab and SCT to induce durable remissions in four out of nine patients [116].

Due to the low frequency of this subtype, it is rarely considered in risk stratification, but for those that do, e.g., the UKALL 2003 study, *TCF3-HLF* is uniformly stratified as high-risk [74].

### 5.13. iAMP21

Intrachromosomal amplification of chromosome 21 (*iAMP21*), a complex chromosomal abnormality, defines a novel cytogenetic subgroup of B-ALL with an unusual mechanism of chromothripsis behind its formation [118]. Patients with iAMP21 tend to be older (with median age 9 years), and they usually present with a low white cell count (median of 5 × 10^9^/L) [119,120]. *iAMP21* is rare, accounting for 1% of childhood ALL. It is best detected using the *RUNX1* FISH probe. As the Ma-Spore study group does not use FISH, we have not consistently found *iAMP21* in our patient group.

Although patients with *iAMP21* were more likely to be MRD-positive at EOI [120], interestingly the data on prognostic impact of MRD in these patients are conflicting thus far. The Berlin Frankfurt Munster group found that *iAMP21* patients who were MRD-positive had an inferior outcome compared with MRD-negative patients [121], whereas results from COG suggested that MRD was generally not of prognostic relevance, with the exception of a subgroup of SR patients [120]. However, trials uniformly found that patients with *iAMP21* fared poorly when treated on a standard-risk backbone and this was abrogated by intensification of treatment on a high-risk backbone (EFS 29% to 78%, OS 67% to 89% in the UKALL trial group) [119,120,122].

## 6. *IKZF1* Deletion and Interactions with Genetic Subtypes

Alteration in the *IKZF1* gene, which regulates both B and T lymphoid differentiation, has emerged as an important prognostic factor in ALL. Somatic deletions of the *IKZF1* gene (*IKZF1^del^*) confer a significantly worse outcome for ALL [9,123,124,125,126]. The availability of the MRC Holland multiplex ligation probe-dependent amplification (MLPA), which is easy to use and affordable, has democratized screening for deletions of *IKZF1^del^* [9]. Using the MLPA kit, *IKZF*1*^del^* patients in MS2010 were upstaged to the next higher risk group, and thus treatment intensity level. This upstaging, together with use of imatinib in Ph ALL-positive cases, lowered the 5-y CIR from 30% to 8% and improved 5-year OS from 70% to 92%.

Somatic *IKZF1^del^* occurs in ∼15% in pediatric ALL cases [4,127]. Clinically, *IKZF1^del^* is typically associated with older age at diagnosis, higher WBC, and higher EOI MRD [9,123,124,125,126]. Whole-gene deletions are more prevalent among children aged 1–9 years old with lower WBC counts (median of 7.9 × 10^9^ L), as compared with intragenic deletions [128,129,130].

Overall, *IKZF1^del^* confers an unfavorable outcome [9,125,131,132,133]; 5-y EFS reaches as low as 39% (vs. 73% for *IKZF1*-neg patients, *p* < 0.0001) [124], 8-year OS as low as 56% (vs. 91.0% for *IKZF1*-neg patients, *p* < 0.001) [134], and 5-year CIR as high as 73% (vs. 25% for *IKZF1*-neg patients, *p* < 0.0001) [124]. This was also true for children with Down syndrome and ALL, where *IKZF1^del^* conferred a dismal 6-year EFS of only 21% [131].

*IKZF*1*^del^* is over-represented in high-risk subtypes (Table 1): 45% of *Ph* ALL, 60% Ph-like, and 88% of *CRLF2*. Even in the HRG group, *IKZF1^del^* conferred a further poorer outcome. For example, for Ph ALL patients, before the era of TKIs, *IKZF1^del^* conferred worse prognosis (4-year DFS of 55.5 ± 9.5% for *IKZF1^del^* vs. 75.0 ± 21.7% no-*IKZF1^del^*) [135]. Ph-like ALL patients fared worse with additional *IKZF1^del^* (5-year EFS 48.6 ± 7.0% vs. 71.7 ± 8.0%) [77], although another study found that presence of *IKZF1^del^* did not seem to confer a higher relapse risk [136].

For relapsed ALL, *IKZF1^del^* also confers an inferior outcome even after stem cell transplantation [137,138]. In the UKALL relapse protocols, patients with *IKZF1^del^* had a rather dismal 5-year OS of 30% compared with 60% for their *IKZF1*-neg counterparts [137,138].

**Figure 6:**
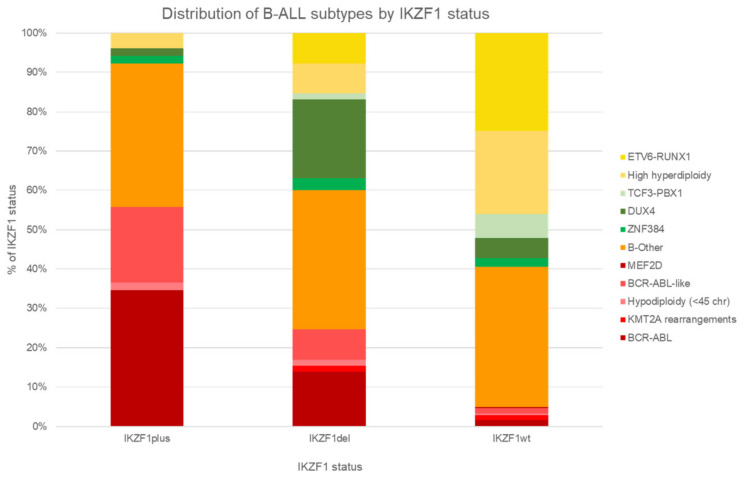
Distribution of subtypes co-occurring with *IKZF1^del^*. The proportions of molecular subtypes co-occurring with *IKZF1^del^* and *IKZF1^plus^* are plotted and compared against subtypes with wild type *IKZF1* (*IKZF1*-neg) for patients treated in the Malaysia-Singapore ALL trial group cohort. *IKZF1^plus^* has additional deletions of *PAX5*, *CDKN2A/B*, or *PAR1* in addition to *IKZF1^del^*. Favorable-risk subtypes are in shades of yellow, intermediate-risk subtypes are in shades of green, and high-risk subtypes are in shades of red. B-others is depicted in orange. There is a greater proportion of high-risk subtypes co-occurring with *IKZF1^del^* and *IKZF1^plus^*, in particular *BCR-ABL*, and a smaller proportion of low-risk subtypes (*p* < 0.001).

Stanulla et al. showed that additional deletions of *PAX5*, CDKN2A/B, and PAR1 in addition to *IKZF1^del^*, which they defined as *IKZF1^plus^*, conferred an even poorer outcome than *IKZF1^del^* alone, especially in MRD-IR and MRD-HR groups. In MS2003/2010, the prevalence of HRG increased dramatically in *IKZF1^del^* and *IKZF1^plus^* compared with *IKZF1*-neg patients (Figure 6). Interestingly, a higher proportion of younger children have *IKZF1*^plus^ (<10 years: 53.0% for *IKZF1^del^* vs. 61.9% for *IKZF1^plus^*, *p* < 0.001) [132].

In MS2003, *IKZF*1*^del^* within the *PAX5*alt group conferred an extremely high risk of relapse (5-year CIR 80%). These relapses in *PAX5*alt tend to be late (2–3 years from diagnosis) and extramedullary in nature (CNS/testicular). This adverse effect of co-deletion of *IKZF1^del^*/*PAX5*alt seemed to be reversible with intensified therapy in MS2010 [9]. The presence of *IKZF1^del^* with *PAX5*alt may explain why *PAX5alt* patients in COG AALL0232 and adult ALL (ECOG/CALGB/SWOG) did poorly [3].

However, not all subgroups with *IKZF1^del^* require treatment intensification. Subgroup analyses by us and others have found that accompanying favorable cytogenetics, e.g., *ETV6-RUNX1*, high hyperdiploidy, and IRG with good outcome such as *TCF3-PBX1*, or *DUX4*/*ERG^de^*^l^, attenuates the negative impact of *IKZF1^del^* [9,63]. *IKZF1^del^* occurs in 7% of *ETV6-RUNX1*, 6% of hyperdiploid, 3% of *TCF3-PBX1*, and 28% of *DUX4*. In these four genetic subtypes, *IKZF1^del^* does not appear to affect clinical outcome. In patients with other subtypes who are EOI MRD-negative, *IKZF1^del^* also does not confer any adverse outcome. Taken together, *IKZF1^del^* conferred poorer outcome in three main groups: IRG (except *TCF3-PBX1* and *DUX4*), HRG, and those who are EOI MRD-positive.

Mechanistically, the adverse molecular mechanisms of *IKZF1^del^* remain incompletely understood. *IKZF1^del^* is found to decrease differentiation and increase focal adhesion proteins that result in cell mislocalization in the extravascular niche [139]. Of interest, Churchman et al. demonstrated reversal of this phenomenon in *IKZF1*-aberrant *BCR-ABL* ALL by treatment with retinoid receptor agonists, thereby suggesting a possible therapeutic avenue for *IKZF1^del^* leukemias [140].

Recently, germline *IKZF1* has also been characterized as a leukemia predisposition gene, where adverse germline *IKZF1* variation has been found in familial pediatric ALL and occurs in approximately 1% of B-ALL patients [141].

## 7. T-ALL

Although T-cell ALL seems more genetically diverse than B-cell ALL [10,142,143], no recurrent genetic aberration in T-ALL confers a distinctly different prognostic outcome. The majority of genetic lesions in B-cell ALL and T-cell ALL are mutually exclusive. Only a few lesions can be found in both, namely *MLL* and *BCR–ABL1* rearrangements [5]. T-ALL can be divided into biological subgroups either by transcription factor oncogenes or by dysregulated functional pathways [10,142]. The most commonly mutated amongst these various transcription factors include *TAL1*, *TAL2*, *TLX1* (also known as *HOX11*), *HOXA*, *LMO1*, *LMO2/LYL1*, and *NKX2-1* [5,10,142]. However, unlike B-ALL subtypes, no distinct T-ALL genetic alterations have been identified that are reproducibly associated with clinical outcomes. Therefore, for the purposes of this review, associations of T-ALL with clinical characteristics will not be addressed for every single genetic subtype, other than those with known relevance.

ETP ALL, or early T-cell precursor ALL, is a distinct form of leukemia characterized by reduced expression of T-cell markers (CD1a, CD8, and CD5) and aberrant expression of myeloid or stem-cell markers [144]. The gene expression profile of ETP-ALL is similar to that of hematopoietic stem cells, suggesting that ETP-ALL may lie closer along the spectrum of immature leukemias rather than true T-ALL, which is mature [4]. This immaturity of ETP-ALL mimics sensitivity to venetoclax in AML, also through BCL-2 dependence [145].

### 7.1. T-ALL Interaction with Age and WBC

Compared with patients with B-cell ALL, T-cell ALL patients are generally older, with a median presenting age of 9 years [10,146]. More than 50% of T-ALL also present with hyperleukocytosis, with a median count of 76 × 10^9^/L [146,147]. However unlike for B-ALL, the prognostic importance of age, presenting WBC, and NCI criteria in T-ALL is limited. Within T-ALL subtypes, *SIL-TAL1* patients have no age preponderance, but had a higher WBC count at presentation (median 174,000/µL) [148]. Children with ETP ALL tended to have lower WBC at presentation, but there was generally no difference in age presentation compared with non-ETP T-ALL [149]. The clinical characteristics of most of the other T-ALL genetic subtypes are not well defined.

### 7.2. T-ALL Interaction with MRD and Outcomes

Compared with B-ALL, MRD kinetics in T-ALL is much slower. A large percentage of T-ALL patients have detectable EOI MRD. However, this high proportion of EOI MRD positivity in T-ALL is prognostically less significant compared with B-ALL. T-ALL outcomes remain favorable as long as they have low-level or undetectable EOC MRD [34,150]. Therefore, while a later MRD timepoint (i.e., consolidation) most effectively identifies HR T-ALL patients, the earlier end-induction timepoint is useful for identifying lower-risk patients who can receive de-intensified therapy [74,151].

With contemporary therapy, T-ALL has achieved outcomes similar to that of B-ALL. Intensifying induction with dexamethasone, Protocol Ib in BFM, HDMTX, and Capizzi methotrexate-asparaginase during interim maintenance has improved the outcomes of patients [10]. Also historically associated with a poorer outcome, ETP-ALL now has similar survival to conventional T-ALL with modern risk-adapted therapy [149,152]. However, notably, relapsed T-ALL is notoriously difficult to salvage, especially because T-ALL becomes highly refractory to chemotherapy upon relapse [153,154,155,156]. Historical reinduction remission rates in relapsed T-ALL are estimated to be poor at 30% to 40% [157].

Unlike B-ALL where there are various forms of effective immunotherapy, T-ALL has fewer effective options currently [157]. There is thus a need for a better way to treat relapsed or refractory cases. The involvement of *JAK*-*STAT* and *PRC2* pathways in ETP-ALL suggests that *JAK* inhibition and/or chromatin-modifying agents may be therapeutically useful. Despite promising preclinical studies inhibiting NOTCH signaling by g-secretase inhibitors, severe GI toxicities and lack of cytotoxic antitumor responses still limit their direct translation into patient benefit. More recently, preclinical studies have shown that dasatinib, an *ABL*-class inhibitor usually given for treatment of *BCR-ABL1* ALL, is surprisingly effective in a large proportion of pediatric T-ALL cases in vivo and in vitro, by which the drivers of drug sensitivity are LCK-dependent and *ABL*-independent [158]. In addition, venetoclax, a bcl2 inhibitor, when combined with chemotherapy may improve response and survival outcomes in T-ALL.

## 8. Conclusions

Exciting progress in genomic sequencing has greatly refined the genetic taxonomy of ALL. With these new genetic entities clinically characterized, each with its own unique prognostic and therapeutic vulnerability, we can refine ALL risk stratification beyond MRD and NCI criteria. Understanding the unique clinical characteristics underpinning each subtype can aid the clinician in the management of ALL. Further, a reliable and comprehensive molecular identification of ALL genetic aberrations, including the ability to detect rare subtypes, is critical for integrated risk-adapted therapy. Ultimately, identifying the complete constellation of genetic aberrations paves the way for potential therapeutic targeting and precision medicine in childhood ALL. Taken together, better and more accurate risk assignment will enable improved cures for ALL, with lesser side effects.

## Figures and Tables

**Table 1:** Overview of B-ALL subtypes and summary of clinical characteristics of individual subtypes based on Malaysia-Singapore 2003 and 2010 trials.

Risk Group	Subtype	Proportion of B-ALL	Median Age at Presentation, Years (Range)	Median Presenting WBC, ×10^9^/L (Range)	Proportion of MRD-Negative at EOI, % (No of Patients)	Proportion of MRD-Negative at EOC, % (No of Patients)	Proportion with *IKZF1^del^*, % (No of Patients)	Interaction with *IKZF1^del^*	5-y CIR, % (Range)	5-y OS, % (Range)
FRG	*ETV6-RUNX1*	20%	4.0 (1.6–14)	12 (1–285)	76% (52/86)	98% (60/61)	7% (5/70)	Possible attenuating	5.2 (1.3 to 13)	100%
	Hyperdiploidy	24%	3.7 (1.4–12.2)	9 (1–608)	54% (46/85)	92% (58/63)	6% (4/66)	Possible attenuating	5.5% (1.7 to 12.6)	98.8% (91.8 to 99.8)
IRG	*TCF3-PBX1*	5%	4.8 (1.5–15.6)	56 (6–224)	58% (11/19)	94% (17/18)	0% (0/18)	Possible attenuating	5.6% (0.3 to 23.1)	94.4% (66.6 to 99.2)
	*DUX4*	14%	9.8 (2.4–16.7)	10 (2–142)	22% (11/50)	82% (33/40)	28% (13/46)	Possible attenuating	8.9% (2.8 to 19.5)	97.8% (85.3 to 99.7)
	*ETV6-RUNX1*-like	2%	2.7 (1.4–12.6)	69 (1–278)	57% (4/7)	83% (5/6)	62% (5/8)	None	12.7% (0.5 to 45.3)	88.9% (43.3 to 98.4)
	*ZNF384*	5%	6.8 (2.1–15.7)	37 (5–140)	18% (3/17)	77% (10/13)	19% (3/16)	None	6.3% (0.4 to 25.5)	93.3% (61.3 to 99.0)
	*ZNF384*-like	1%	5.1 (2.5–7.7)	76 (62–90)	50% (1/2)	50% (1/2)	0% (0/1)	None	-	-
	*NUTM1*	1%	2.4 (0.8–11.3)	33 (11–53)	100% (3/3)	100% (1/1)	0% (0/3)	None	0.0%	100.0%
	*PAX5*alt	10%	3.9 (0.7–17.4)	24 (2–509)	39% (12/31)	89% (25/28)	28% (9/32)	Poorer prognosis, *IKZF^plus^*	18.1% (6.3 to 34.7)	92.8% (73.7 to 98.2)
	*PAX5*-P80R	1%	5.7 (5.0–6.3)	3 (2–5)	0% (0/2)	100% (1/1)	0% (0/2)	Poorer prognosis, *IKZF^plus^*	-	-
	B-Others	7%	5.1 (0.6–13.0)	8 (1–124)	45% (10/22)	89% (16/18)	0% (0/21)	None	20.7% (7.3 to 39.0)	94.1% (65.0 to 99.1)
	*IGH-CEBPE*	<1%	3.8 (3.8–3.8)	32 (32–32)	0 (0/1)	100% (1/1)	0% (0/1)	None	-	-
HRG	Ph (*BCR-ABL1*)	2%	10.6 (2.7–15.2)	180 (7–708)	44% (4/9)	83% (5/6)	44% (4/9)	Poorer prognosis	37.5% (7.2 to 69.4)	75.0% (31.5 to 93.1)
	Ph-like (*BCR-ABL*-like)	2%	8.0 (2.4–14.1)	22 (4–518)	12% (1/8)	60% (3/5)	60% (3/5)	Poorer prognosis	37.5% (6.9 to 69.8)	75.0% (31.5 to 93.1)
	*MLL (KMT2A)*	3%	0.5 (0.2–3.4)	42 (5–247)	11% (1/9)	43% (3/7)	0% (0/10)	None	54.3% (16.7 to 81.2)	64.8% (25.3 to 87.2)
	Hypodiploidy	1%	15.1 (13.8–16.4)	9 (6–12)	0% (0/1)	0% (0/1)	0% (0/1)	None	50.0% (0.0 to 96.0)	50.0% (0.6 to 91.0)
	Near-haploidy	1%	6.6 (4.3–8.3)	26 (4–246)	100% (3/3)	100% (3/3)	0% (0/2)	None	50.0% (0.0 to 96.0)	50.0% (0.6 to 91.0)
	*MEF2D*	1%	11.0 (4.9–12.4)	7 (5–11)	100% (4/4)	100% (3/3)	0% (0/4)	None	0.0%	100.0%
	*HLF-r*	<1%	5.2 (5.2–5.2)	183 (183–183)	0% (0/1)	N.A	0% (0/1)	None	-	-
	*CRLF2*	3%	8.3 (3.0–17.3)	59 (11–145)	22% (2/9)	88% (7/8)	80% (8/10)	Poorer prognosis	20.0% (2.6 to 49.2)	59.1% (16.0 to 86.0)

**Table 2:** EOI MRD response in favorable-risk genetic risk subtypes. *ETV6-RUNX1* has the second most rapid MRD response among all the genetic subgroups after *TCF3-PBX1*.

EOI MRD <0.01%	Total 15 (Day 19)	Total 16 (Day 15)	COG	UKALL 2003	MS2003/2010
*ETV6-RUNX1*	58%	54%	90%	73%	76%
Hyperdiploidy	44%	31%	80%	52%	54%
